# Intermittent Hypoxia Enhances THP-1 Monocyte Adhesion and Chemotaxis and Promotes M1 Macrophage Polarization via RAGE

**DOI:** 10.1155/2018/1650456

**Published:** 2018-10-08

**Authors:** Jing Zhou, Wei Bai, Qin Liu, Jian Cui, Wei Zhang

**Affiliations:** Department of Respiratory Medicine, The First Affiliated Hospital of Nanchang University, Nanchang, China

## Abstract

Intermittent hypoxia (IH) that resulted from obstructive sleep apnea (OSA) has been found to be a risk factor of coronary artery disease. IH and the receptor for advanced glycation end products (RAGE) expression are known to activate monocyte/macrophage and associated with atherosclerosis development, while their effects on monocyte adhesion, chemotaxis to the endothelium, and macrophage polarization remain unknown. In the present study, RAGE in THP-1 monocytes was inhibited by shRNA lentiviral particles, followed by exposure to IH. Cell adhesion assay, transwell migration assay, and macrophage polarization assays were performed to study the effects of IH and RAGE. The mRNA and protein expression levels were investigated by RT/real-time PCR and western blot analysis, respectively. We found that IH increased RAGE expression and activated NF-кB signalling in THP-1 monocytes. The results also revealed that IH enhanced the MCP-1-mediated THP-1 monocyte adhesion and chemotaxis and promoted macrophage polarization toward a proinflammatory phenotype, which was mediated by RAGE activity. Additionally, inhibition of chemokine receptor type 2 (CCR2) suppressed the IH-induced monocyte adhesion and chemotaxis. These results demonstrated a potential role of monocyte adhesion, chemotaxis, and macrophage polarization in the development cardiovascular diseases induced by IH and identified that RAGE could be a promising therapeutic target to prevent atherosclerosis in patients with OSA.

## 1. Introduction

Obstructive sleep apnea (OSA) is a sleep disorder characterized by repeated episodes of obstructive apnea and hypopnea during sleep [1]. The patients often present symptoms of daytime sleepiness or altered cardiopulmonary function, which have been reported to be affecting more than 10% of the adult population [2]. Besides these, it increases the risk of numerous clinical conditions including diabetes mellitus, dyslipidemia, and cardiovascular diseases. Moreover, it has been suggested that OSA is closely associated with atherosclerosis and detrimental cardiovascular events [3].

Intermittent hypoxia (IH) has been identified as one of the hallmark characteristics of OSA. The relationship between IH and atherosclerosis has been verified by many* in vivo* studies [4, 5], which showed that IH induced vascular inflammation and atherosclerotic plaques formation in mice. Additionally, clinical studies have reported that IH increases the levels of serum advanced glycation end products (AGE) in people with OSA [6]. Furthermore, a growing body of evidences suggested that the activation of the specific cell-surface receptor for AGE (RAGE) plays a critical role in the development of atherosclerotic lesion [7, 8]. For example, hypoxia-induced inflammation could be attenuated in RAGE-deficient mice [9]. However, the molecular mechanisms underline that these observations remain poorly understood.

Macrophage is the key regulator in all phases of atherosclerosis, from initial fatty streaks formation to vascular lesions. The formation of atherosclerotic plaque is a highly complex process. The adhesion of monocytes to the endothelium and subsequent transmigration through the vascular endothelial wall are central to the development of atherosclerosis [10]. It has been established that the monocytes are attracted and activated by chemokines released from inflamed endothelial cells, especially, the monocyte chemotactic protein-1 (MCP-1), which is abundantly found in atherosclerotic plaques in human [11, 12]. Also, in response to stimulus, the monocytes differentiate and polarize into inflammatory (M1) or anti-inflammatory (M2) macrophages [13]. M1 macrophages play critical roles in diseases progression by inducing an inflammatory state, which is characterized by upregulated expression of proinflammatory mediators, while M2 macrophages contribute to inflammation resolution via secretion of anti-inflammatory cytokines [14]. M1/M2 ratio is believed to be a major determinant of atherosclerotic lesions [13]. It has been showed that RAGE mediates monocyte differentiation and lipids accumulation in macrophages that lead to foam cell formation [15]. However, few study has investigated the possible regulatory role of RAGE in monocyte adhesion, migration, and macrophage polarization in IH, which are required for an improved understanding of the mechanisms that how atherosclerosis develops in patients with OSA.

Therefore, the aims of the present study were to investigate the RAGE expression in monocytes and to elucidate its roles in monocyte adhesion, migration, and macrophage polarization in IH using an* in vitro* THP-1 monocytes model and monocyte-endothelia cell coculture model.

## 2. Materials and Methods

### 2.1. Reagents and Antibodies

Monocyte chemoattractant protein-1 (MCP-1) was purchased from R&D Systems (MN, USA). Primary antibodies for human RAGE, IkB*α*, NF-*κ*B p65, phosphorylated NF-*κ*B p65, CCR2, and GAPDH were purchased from Abnova (Taipei, Taiwan). Alexa 488 conjugated secondary antibody, Alexa Fluor 555 Phalloidin, and DAPI were purchased from Beyotime (Shanghai, China). The siRNA duplexes against human RAGE (ID: 110859) were purchased from Ambion Life Technologies (NY, USA). The RAGE shRNA lentiviral particles and control shRNA lentiviral particles were purchased from Santa Cruz Biotechnology (USA). The CCR2 neutralizing antibody and CCR2 Isotype control were purchased from Santa Cruz Biotechnology (USA). The CCR2 antagonist (C_28_H_34_F_3_N_5_O_4_S, CAS 445479-97-0) was purchased from Merck Millipore (USA).

### 2.2. Cell Culture and Treatment

THP-1 cells (human monocytic leukemia cell line) were purchased from the American Type Culture Collection (ATCC) and cultured in suspension in T-75 culture flasks with RPMI 1640 medium containing 10% fetal bovine serum, 100 U/mL penicillin, 100 *μ*g/ml streptomycin, 1 mM glutamine, 10 mM HEPES, 50 *μ*M *β*-mercaptoethanol, and 1 mM sodium pyruvate. For macrophage polarization experiments, THP-1 monocytes were treated with 10 ng/ml Phorbol 12-myristate 13-acetate (PMA, Sigma Aldrich, USA) for 48 h to differentiate into adherent macrophage-like cells prior to exposure to IH for 24 hours and analysis of macrophage markers. Human vascular endothelial cells (HUVECs) were grown in EGM-2 medium (Lonza, USA). HUVECs at passages 3-7 were used in the experiments. The cells were maintained in a humidified incubator with 5% CO_2_/95% air at 37°C.

### 2.3. RNA Interference (RNAi)

For siRNA tests, THP-1 monocytes were pretreated with NF-kB siRNA or control siRNA using the Cell Line Nucleofector (Lonza, USA) and buffer kit V (Lonza, USA) according to the manufacturer's protocol. After 48 h, treated cells were exposed to IH for 24 h followed by protein samples collection for western blot assays.

To silence the RAGE gene, THP-1 monocytes at a density of 2 × 10^5^ cells/well in a 6-well plate were first suspended in growth media supplemented with 5 *μ*g/mL polybrene and incubated overnight. Then RAGE shRNA lentiviral particles or control shRNA lentiviral particles were added. After 48 h of incubation at 37°C, the viral load was removed by centrifugation. THP-1 monocytes were washed with PBS and cultured in growth media. Successfully transfected THP-1 monocytes were selected by treatment with 10 *μ*g/mL puromycin (Santa Cruz Biotechnology, USA) until all cells in the control flask were confirmed dead.

### 2.4. Conditions of Normoxia and Intermittent Hypoxia

Condition of normoxia or IH was conducted in a specifically designed gas flow chamber modified from In Vivo 400 Hypoxic Work Station (Biotrace, Cincinnati, OH). Treated cells were cultured in condition of normoxia (21% O_2_, 5% CO_2_) or IH (6 cycles of 35 min of hypoxia [0.1% or 5% O_2_ and 5%CO_2_] followed by 25 min of normoxia; CO_2_ was maintained at 5% throughout the exposure). The chamber was placed in a standard humidified incubator at 37°C.

### 2.5. Western Blotting

Protein samples were extracted from THP-1 monocytes according to the method established previously [16]. The cell lysates were collected and protein concentration was determined using standard BCA assay. Equal amounts of protein (20 *μ*g/sample) were subjected to SDS-PAGE (4–10%) followed by electrophoretic transfer to nitrocellulose membranes. In order to block the nonspecific binding, the membranes were incubated with skim milk (5%) for 1 h at room temperature. After that, the blot was incubated with one of the following primary antibodies overnight at 4°C: rabbit anti-RAGE IgG (1:500), anti-phosphorylated NF-*κ*B p65 (1:1000), anti-I*κ*B*α* IgG (1: 1000), anti-CCR2 IgG (1:1000), and anti-GAPDH IgG (1:3000). After that, membranes were incubated with suitable HRP-conjugated second antibody (1:2000) at 37°C for 1 h, followed by washing and reacting with the ECL detection reagents followed by scanning on a densitometer. The signals specific for target proteins on the gel were then analyzed.

### 2.6. Total RNA Extraction and RT-PCR

Total RNA from THP-1 monocytes/macrophages was extracted using the RNeasy Plus Mini kit (Qiagen). The RNA was quantified using Thermo Bio-Mate3 and diluted into 1 mg/mL. Total RNA (0.5 *μ*g) was processed directly to cDNA synthesis using the oligo (dT) 15 primer and SuperScript™ II reverse transcriptase reagents (TaKaRa, Dalian, China) according to the manufacturer's protocol. Real-time PCR was performed using qSYBR Green PCR Kit (Dongsheng, Guangzhou, China). All PCR primers were designed using software PrimerExpress or published sequence data from the NCBI database. The sequences of forward and backward primers are listed in Table 1. All these primers were synthesized from Invitrogen (USA). All reactions were performed in triplicate in an ABI PRISM 7900HT Sequence Detection system. Data are calculated by the 2^−ΔΔCT^ method and are presented as the fold induction to GAPDH compared with THP-1 monocytes/macrophages cultured in normoxia.

### 2.7. Cell Adhesion Assay

THP-1 monocytes were first activated by 20 ng/ml MCP-1 for 12 h. Then 2 × 10^5^ THP-1 monocytes were added to each well containing a monolayer of confluent HUVECs in a 24-well tissue culture plate. THP-1 monocytes and HUVECs cells were incubated at 37°C in normoxia or IH with nonadhered cells removed by gentle washing with warm PBS twice after 30 min. The number of adhered THP-1 monocytes was then counted using the inverted microscope.

### 2.8. THP-1 Monocytes Migration

A transwell migration assay was applied to study the transmigration behaviour of THP-1 monocytes. 24-transwell inserts with pore sizes of 3 *μ*m (Corning, USA) were employed. The lower surface of the insert was coated with gelatin solution (1 mg/mL) and allowed to dry for 15 min before experiments. 10^6^ THP-1 monocytes in 200 *μ*l of serum-free RPMI 1640 were loaded into the upper chamber of the transwell insert. 600 *μ*l RPMI medium containing 10 ng/ml MCP-1 (R&D Systems, USA) was added to the lower chamber. The cells were then allowed to migrate in normoxia or IH for 8 h. The number of cells that had migrated across the membrane to the lower chamber was counted microscopically using a Motic AE30/31 inverted microscope (Motic Co, China).

### 2.9. Immunofluorescence Staining

THP-1 cells were fixed with 4% paraformaldehyde (Beyotime, China) and permeabilized with 0.5% Triton X-100 (Beyotime, China). PBS with 5% BSA was used to block the nonspecific binding sites. Monoclonal anti-NF-*κ*B primary antibody in PBS with 1% BSA was incubated with the sample overnight. After this, cells were washed and incubated for 1 h at room temperature in Alexa 488 conjugated anti-Mouse IgG as secondary antibody. To visualize the F-actin, the samples were incubated with 200 *μ*l of Alexa Fluor 555 Phalloidin at room temperature for 20 mins in the dark. Cell nuclei were stained by incubation with 5 *μ*g/ml DAPI for 5 min. Slides were then visualized with a fluorescence microscope (SP2, Leica, Germany) using x40 objective.

### 2.10. Data Analysis

The results are presented as mean ± SEM for each treatment group in each experiment with all experiments repeated for at least three times. Statistical significance was analyzed by one-way analysis of variance using computer software SPSS (Version 20). The data between different experimental groups were analyzed by Student's Newman-Keuls test and p < .05 was considered statistically significant.

## 3. Results

### 3.1. Intermittent Hypoxia Upregulated RAGE Expression and Activated NF-кB Pathway in THP-1 Monocytes

**Figure 1:**
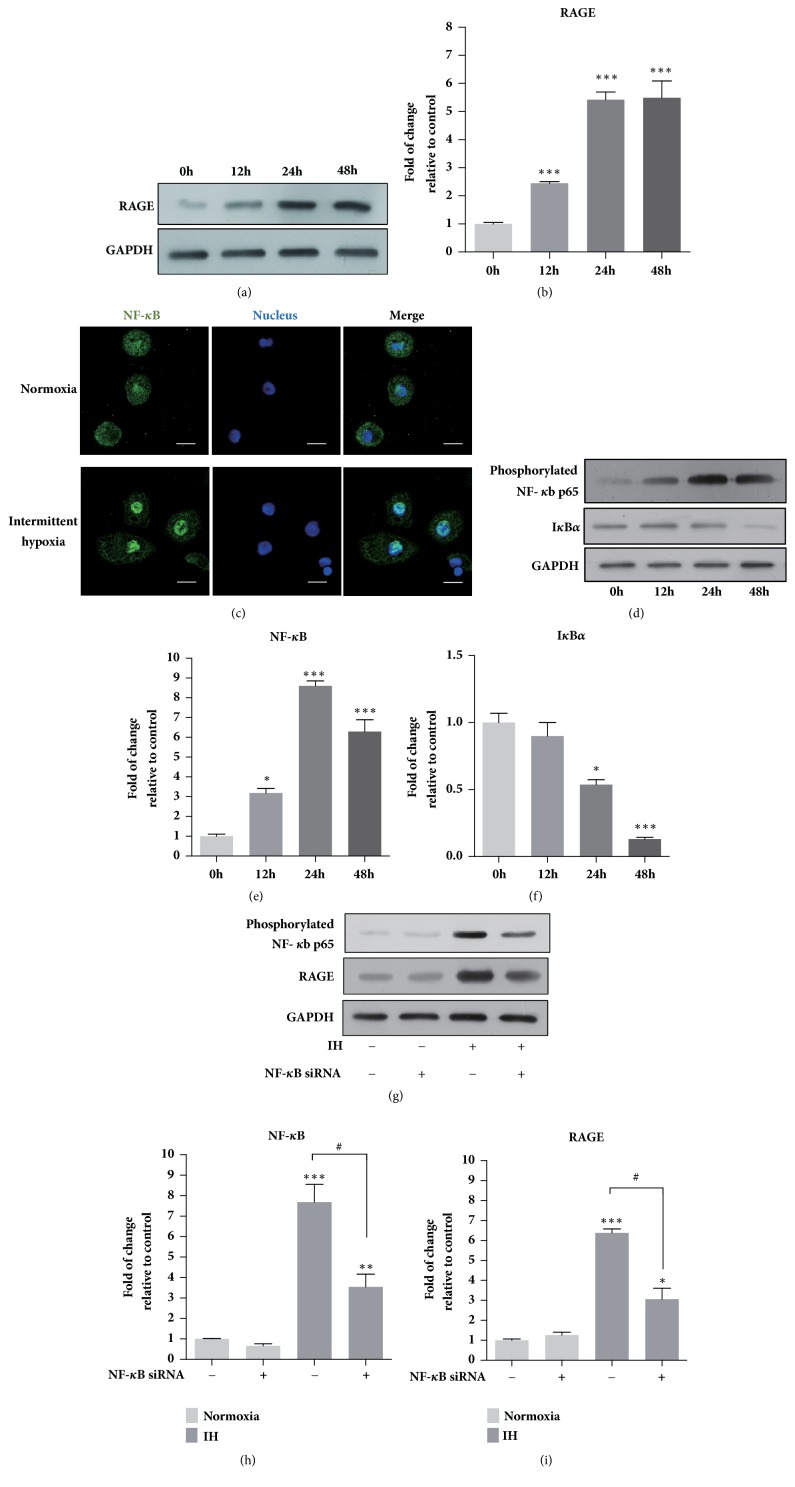
Intermittent hypoxia increased RAGE expression and activates the NF-кB pathway in THP-1 monocytes. (a and b) RAGE expression of THP-1 cells exposed to IH compared to cells in normoxia over a period of 48 h. (c) Representative images of immunofluorescence staining showing the nuclear translocation of NF-кB p65 (green) in THP-1 cells. Scale bars indicate 10 *μ*m. (d–f) Phosphorylated NF-*κ*B p65 and IkB*α* protein expression and quantification of THP-1 cells exposed to IH compared with cells cultured under normoxia over a period of 48 h. (g–i) Phosphorylated NF-*κ*B p65 and RAGE protein expression and quantification of NF-*κ*B siRNA treated cells compared to untreated cells exposed to normoxia or IH. Data were represented as mean + SEM. *∗* represents significant difference compared with normoxic group, *∗*/^#^p<.05, *∗∗*p<.01, *∗∗∗*p<.001.

According to the time course experiment shown in Figures 1(a) and 1(b), in the IH-exposed THP-1 monocytes, the level of RAGE was increased in a time-dependent manner. The expression of RAGE in THP-1 cells was increased by around 3-fold after 12 h in IH. Then RAGE protein level reached a peak value of increase after 24 h incubation and remained at that level for the next 24 h. In addition, after exposure to IH for 24 h, NF-*κ*B translocated and accumulated into the nucleus of THP-1 cells (Figure 1(c)). Also, the protein level of phosphorylated NF-*κ*B p65 was increased while IkB*α* was decreased in a similar time-dependent manner in IH (Figures 1(d)–1(f)) thus confirming the activation of NF-*κ*B signalling. Furthermore, by using NF-*κ*B siRNA to downregulate NF-*κ*B (Figures 1(g) and 1(h)), it was observed that NF-*κ*B siRNA was able to significantly reduce the upregulation of RAGE expression that resulted from IH (Figures 1(g) and 1(i)), which suggested that NF-*κ*B signalling played a positive role in the activation of RAGE in IH.

### 3.2. Knockdown of RAGE Inhibited Adhesion and Chemotaxis of THP-1 Monocytes Exposed to Intermittent Hypoxia

**Figure 2:**
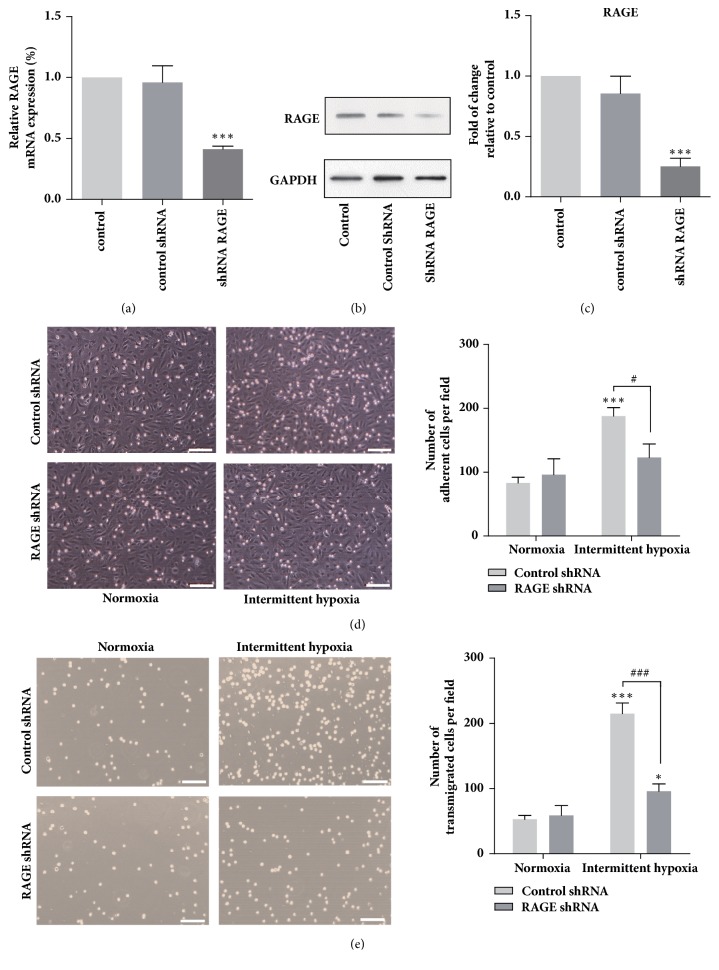
RAGE knockdown inhibited chemotaxis and adhesion of THP-1 cells exposed to intermittent hypoxia. Knockdown of RAGE in THP-1 cells was verified via qRT-PCR (a) and western blotting (b and c). Representative images with quantification of the results for THP-1 cells (d) transwell migration assay and adhesion assay (e) in presence of MCP-1. Data were represented as mean + SEM. *∗* represents significant difference compared with untreated cells under normoxia condition; *∗*^/#^p<.05, *∗∗∗*/^###^p<.001. Scale bars indicate 100 *μ*m.

To investigate the role of RAGE in THP-1 cells, lentiviral shRNA targeting RAGE was introduced. We first verified that the RAGE shRNA lentiviral particles significantly inhibited the mRNA expression (Figure 2(a)) and protein expression levels of RAGE in THP-1 cells compared with the cell treated by control shRNA (Figures 2(b) and 2(c)). These results demonstrated that the RNAi strategies were effective. It has been established that the adhesion of monocytes to the vascular endothelial cells triggered by MCP-1 contributes to the early development of atherosclerosis [10]. In addition, monocyte chemotactic protein-1 (MCP-1) is responsible for the recruitment of monocytes, which is abundantly present in macrophage-rich atherosclerotic plaques [11, 12]. Therefore, the adhesion of monocytes to the endothelia activated with MCP-1 and the chemotaxis of treated THP-1 monocytes toward MCP-1 in IH was examined. To investigate the adhesion of THP-1 cells to endothelium, the cells were first stimulated by MCP-1 and then adhesion assay was conducted under normoxia or IH. We observed that IH exposure led to an approximately 90% increase in the number of the THP-1 cell adhered to endothelial cells while RAGE knockdown markedly reduced the IH-induced upregulation of cell adhesion (Figure 2(d)). It is further showed in Figure 2(e) that IH promoted the chemotaxis of THP-1 cells toward MCP-1 with the number of cells successfully migrated through the transwell membrane increased by approximately 3-fold compared with cells in normoxia. Furthermore, knockdown of RAGE reduced the number of migrated cells exposed to IH by around 55%.

### 3.3. Intermittent Hypoxia Promoted CCR2 Expression via RAGE, Which Mediated Adhesion and Chemotaxis of THP-1 Monocytes

**Figure 3:**
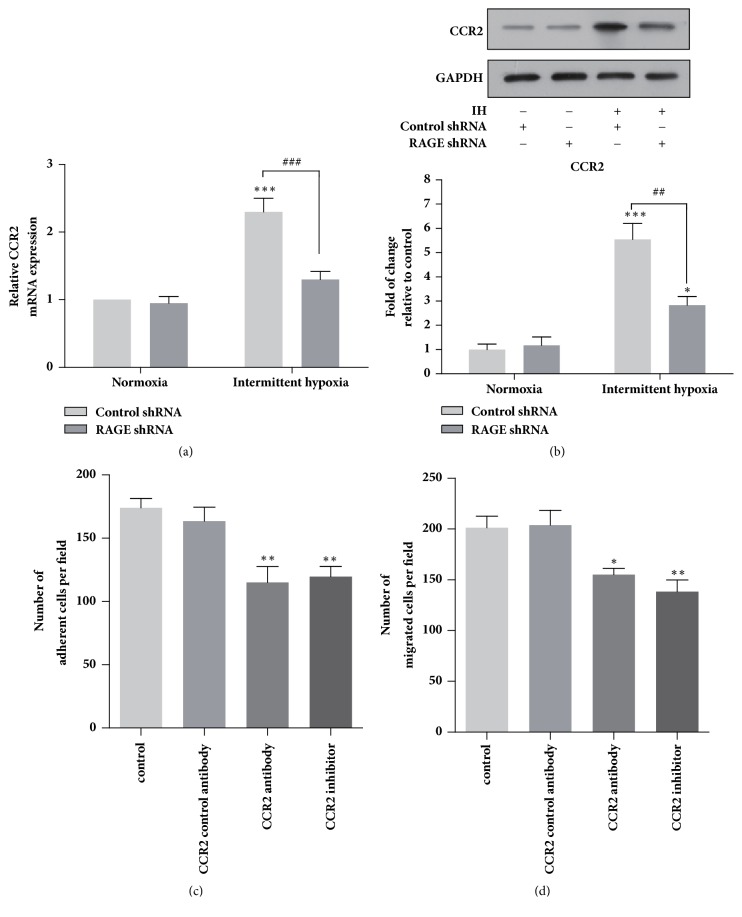
Intermittent hypoxia promoted CCR2 expression via RAGE, which mediated chemotaxis and adhesion of THP-1 cells. Expression levels of CCR2 in THP-1 cells exposed to normoxia and intermittent hypoxia determined by qRT-PCR (a) and western blotting (b). The effects of CCR2 neutralizing antibody (10 *μ*g/m) and CCR2 inhibitor (10 nM) on (c) the adhesion of THP-1 monocytes to HUVECs and the cell transwell migration (d). Data were represented as mean + SEM. *∗* represents significant difference compared with control group under normoxia condition; *∗*p<.05, *∗∗*/^##^p<.001, *∗∗∗*/^###^p<.001.

Chemokines and their receptors are responsible for the attraction, chemotaxis, and adhesion of monocytes that trigger the development of atherosclerosis [10]. Among the chemokine receptors, chemokine receptor type 2 (CCR2) is expressed in almost all circulating monocytes, which plays an essential role for the chemotaxis of monocytes toward the inflamed sites. We observed that both mRNA and protein expression level of CCR2 were significantly increased in THP-1 monocytes when cultured under IH (Figures 3(a) and 3(b)). Moreover, knockdown of RAGE markedly reduced IH-induced CCR2 expression in THP-1 cells. In order to determine the role of CCR2 in IH-induced monocyte adhesion and migration, we further treated the THP-1 cell with CCR2 neutralizing antibody (10 *μ*g/m) or CCR2 inhibitor (10 nM) for 1 h prior to chemotaxis and adhesion assays. As showed in Figures 3(c) and 3(d), the monocyte adhesion to HUVECs promoted by IH was significantly downregulated by blocking CCR2 with CCR2 neutralizing antibodies and inhibitor. Similarly, the migration of THP-1 cells was reduced by approximately 30 and 42% with CCR2 antibodies and inhibitor treatment, respectively, compared with cells only exposed to IH.

### 3.4. RAGE Plays an Indispensable Role in THP-1 Macrophages Polarization in Intermittent Hypoxia Condition

**Figure 4:**
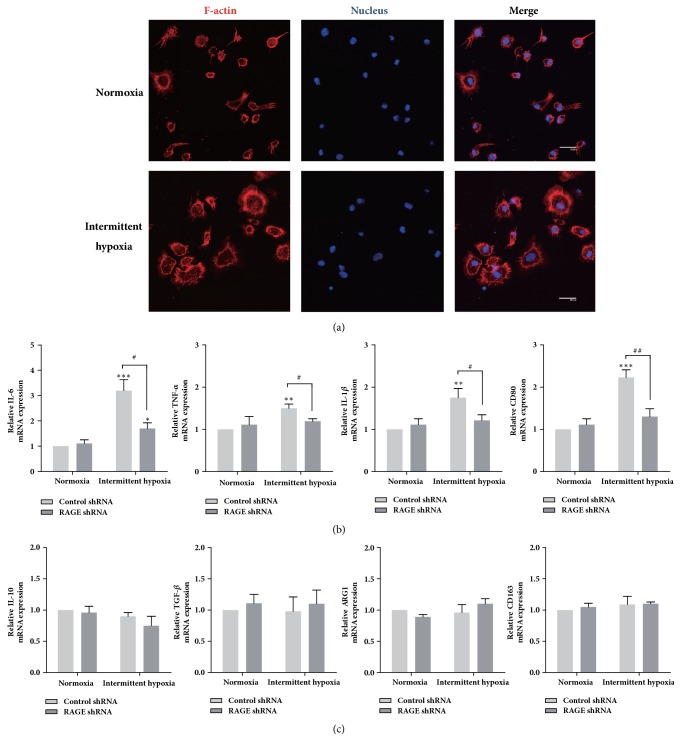
Intermittent hypoxia polarizes macrophages to an M1 phenotype, which was mediated by RAGE. (a) Morphology of THP-1 macrophage cultured in normoxia or IH. F-actin cytoskeleton of the cells were labelled with phalloidin (red) and nuclei stained with DAPI (blue). Scale bar: 20 *μ*m. (b) mRNA expression levels of M1 expression markers. (c) mRNA expression levels of M2 expression markers. *∗* represents significant difference compared with control group under normoxia condition; *∗*p<.05, ^##^p<.001, *∗∗∗*/^###^p<.001.

M1 macrophage releases higher level of TNF-*α*, IL-1*β*, IL-6 with higher expression of clusters of differentiation CD80, while M2 macrophage expresses higher level of IL-10, TGF-*β*, arginase 1 (ARG1), and CD163. In order to investigate the effect of RAGE on macrophage polarization, THP-1 monocytes were first treated with PMA, which activates protein kinase C (PKC) and induces macrophage differentiation [17], to acquire phenotypic and functional characteristics that resemble those of primary macrophages. As showed by the representative images of THP-1 macrophage (Figure 4(a)), there were dramatic changes in cell morphology after exposure to IH, which caused the cells to spread and flatten into a round and pancake-like shape suggesting the maturation of THP-1 macrophages. Our qPCR results also showed that IH substantially and significantly enhanced the expression of M1 markers in THP-1 macrophages. Most significantly, IL-6 mRNA expression was increased by approximately 3.2-fold following IH. TNF-*α*, IL-1*β*, and CD80 expression was also upregulated by around 40%, 75%, and 120%, respectively, versus normoxia group (Figure 4(b)). However, we did not observe any significant changes in the expression of M2 macrophage markers (Figure 4(c)). Furthermore, as shown in Figure 4(b), IH-induced M1 polarization was markedly attenuated by knockdown of RAGE. Additionally, the expression of IL-10 mRNA level was slightly decreased in RAGE shRNA-treated cells, although this effect was not statistically significant (Figure 4(c)).

## 4. Discussion

In spite of the importance of OSA in cardiovascular diseases development, the mechanisms of IH-induced atherosclerosis were not fully understood. There have been growing evidences to support the hypothesis that IH induces atherosclerosis* in vivo* [5, 18, 19]. Previous* in vitro* studies have also revealed that IH activates macrophage and affects macrophage polarization. For example, IH induces lipid-laden macrophages (foam cells) formation in murine macrophages by activating the IKK-*β*-dependent NF-*κ*B pathway [20]. In addition, IH leads to significant increase in IL-6 expression in macrophages and promotes M1 macrophage polarization [21], which reveal the mechanisms by which OSA enhances inflammation and fibrosis in patients with fatty liver disease, while it has been shown that tumor-associated macrophages (TAMs) exhibit reductions in M1 macrophage markers in IH-exposed tumors [22]. Further, our results indicate that IH enhances monocyte adhesion and chemotaxis and promotes M1 macrophage polarization, which unravelled the relation between OSA and monocyte/macrophage recruitment and activation.

Firstly, we revealed that IH exposure promoted the activation of RAGE in THP-1 monocytes. This is in line with the report that AGEs level is increased in OSA condition, which enhances the expression and activation of RAGE [6]. RAGE activates many downstream signalling pathways, among which NF-*κ*B is known as the most critical one [23]. As expected, IkB proteins, as the key inhibitor of the NF-*κ*B pathway, were downregulated and NF-*κ*B activity was increased (Figures 1(c) and 1(d)). Additionally, it has been suggested that NF-*κ*B promotes the transcription of some inflammation factors including the RAGE [24]. Thus we treated the THP-1 cells with NF-*κ*B siRNA and observed that inhibition of NF-*κ*B significantly reduced IH-induced upregulation of RAGE expression at protein levels. Therefore, these results demonstrated that NF-*κ*B pathway played an important role in IH-induced RAGE upregulation in THP-1 monocytes.

It has been documented that IH exposure induces foam cell formation in the THP-1 cells [20]. In addition, monocytes adhesion and transendothelial migration paly an indispensable role in the foam cell formation* in vivo*. How monocytes adhesion and migration would be altered by IH and the key regulators in this process remain largely unknown. In this study, we demonstrated that IH dramatically promoted the adhesion of THP-1 cells to endothelia cells. In addition, chemotaxis ability of THP-1 cells was significantly enhanced by IH. Moreover, we revealed that RAGE played a central role in the IH-induced THP-1 cells adhesion and migration. These results suggested that monocytes exposed to IH could be more easily adhered to endothelial cells and transmigrate into the subendothelium, which would directly trigger the formation of atherosclerotic plaque in OSA patients. CCR2, which is expressed in almost all circulating monocytes, regulates the chemotaxis of monocytes and the pathogenesis of various inflammatory diseases [25]. It was intriguing for our study to unravel the increase of CCR2 expression and its essential role in regulating the adhesion and chemotaxis of THP-1 cells exposed to IH. These findings are consistent with the report that CCR2 gene expression and macrophage infiltration in carotid body were increased in rat exposed to chronic intermittent hypoxia [26].

The leukocyte response to IH* in vitro* was not restricted to cell adhesion and migration. Atherosclerotic lesions are also associated with an increase of M1 macrophages expressing proinflammatory markers in the atherosclerotic plaque [27]. We observed the proinflammatory M1 macrophage polarization in IH. This finding provides the evidence for how IH promotes inflammation via enhancing the formation of activated M1 macrophages in patients with OSA. Previous studies have shown that RAGE activation promotes macrophages to secret proinflammatory cytokines [28], such as IL-1*β*, IL-6, and TNF-*α*, while the role of RAGE on macrophage polarization remains unknown. In this study, we further found that knockdown of RAGE significantly attenuated gene expression of M1 markers of THP-1 macrophages exposed to IH. Therefore, our data proved that IH induced M1 macrophage polarization, which was mediated by RAGE signalling.

The present study indicated that IH enhanced monocytes adhesion and chemotaxis and promoted macrophage polarization toward a proinflammatory M1 phenotype, which was mainly mediated by RAGE activity. These results strongly suggest an important role of RAGE in the cardiovascular diseases of severe OSA patients and provide a promising strategy to prevent atherosclerosis development in patients with OSA.

## Figures and Tables

**Table 1:** Forward and backward sequences of the primers used in this study.

Gene	Forward	Reverse	Genbank ID
RAGE	CTC GAA TGG AAA CTG AAC AC	CTG GTA GTT AGA CTT GGT CTC	177
CCR2	AGT GCT TCG CAG ATG TCC TT	TAG TTC CCA AGT TGC CTG GT	729230
IL-1*β*	TGTGCAAGTGTCTGAAGCAGC	TGGAAGCAGCCCTTCATCTT	3553
IL-6	CTGTTGACAAGCAATGAGACGATGAGG	GAGGATACCACTCCCAACAGACC	3569
IL-10	GCC TAA CAT GCT TCG AGA	TGA TGT CTG GGT CTT GGT TC	3586
Nf-*κ*B	CTGGTGATCGTGGAACAGCC	CAGAGCCTGCTGTCTTGTCC	4790
TNF-*α*	ACA ACC TTC TTG CAG CTC CTC	TGACCCATACCCACCATCAC	7124
TGF-*β*	GGACACCAACTATTGCTTCAG	TCCAGGCTCCAAATGTAGG	7040
CD80	TGGTGCTGGCTG GTCTTTC	CTGTGCCACTTCTTTCACTTCC	941
ARG-1	TACTAGGAAGAAAGAAAAGGCCAATTC	GTAGCCCTGTTTTGTAGATTTCTTCTGT	383
CD163	CCAGTCCCAAACACTGTCCT	ATGCCAGTGAGCTTCCCGTTCAGC	9332
GAPDH	GGC ATG GAC TGT GGT CAT GAG	TGC ACC ACC AAC TGT TAG C	2597

## Data Availability

The data used to support the findings of this study are included within the article.
